# Screening for Safe and Efficient *Monascus* Strains with Functions of Lowering Blood Lipids, Blood Glucose, and Blood Pressure

**DOI:** 10.3390/foods14050835

**Published:** 2025-02-28

**Authors:** Chuling Liu, Li Cheng, Mingtian Yang, Zhengli He, Yanan Jia, Li Xu, Yuansong Zhang

**Affiliations:** College of Sericulture, Textile and Biomass Science, Southwest University, Chongqing 400715, China; 15572379169@163.com (C.L.); 18203011762@163.com (L.C.); yangmingtian2000@126.com (M.Y.); ren630333151@163.com (Z.H.); jiayanan0904@swu.edu.cn (Y.J.); mulberry@swu.edu.cn (L.X.)

**Keywords:** *Monascus*, hyperlipidemia, hyperglycaemia, hypertension, EWM-TOPSIS

## Abstract

*Monascus* is a fungus widely used in food fermentation. This study employed microbial technology, combined with microscopic morphological observations and ITS sequence analysis, to isolate, purify, and identify 10 strains of red yeast mold from various *Monascus* products. After the HPLC detection of metabolic products, the M8 strain containing the toxic substance citrinin was excluded. Using the EWM-TOPSIS model, the remaining nine safe *Monascus* strains were evaluated for their inhibitory activities against pancreatic lipase, α-glucosidase, α-amylase, and the angiotensin-converting enzyme. The M2 strain with the highest comprehensive scores for lowering blood sugar, blood lipids, and blood pressure was selected. Its fermentation product at a concentration of 3 mg/mL had inhibition rates of 96.938%, 81.903%, and 72.215%, respectively. The contents of the blood lipid-lowering active substance Monacolin K and the blood sugar and blood pressure-lowering active substance GABA were 18.078 mg/g and 5.137 mg/g, respectively. This strain can be utilized for the biosynthesis of important active substances such as Monacolin K and GABA, as well as for the fermentation production of safe and effective functional foods to address health issues like high blood lipids, high blood sugar, and high blood pressure in people. This study also provides insights into the use of natural fungi to produce healthy foods for combating chronic diseases in humans.

## 1. Introduction

The term “triple H” refers to hyperlipidemia, hyperglycemia, and hypertension [1], which are prevalent chronic conditions often interrelated and collectively constitute risk factors for cardiovascular disease. Hyperlipidemia denotes elevated lipid levels in the bloodstream, primarily encompassing high cholesterol and high triglycerides. This condition tends to promote plaque formation along blood vessel walls, contributing to arteriosclerosis and elevating the likelihood of cardiovascular ailments [2]. Hyperglycemia signifies heightened glucose concentrations in the blood. Prolonged hyperglycemia characterizes diabetes and can precipitate various complications, including cardiovascular disease, kidney disorders, retinopathy, and neurological ailments [3]. Hypertension is characterized by sustained high blood pressure levels, typically defined as systolic pressure exceeding 140 mm Hg or diastolic pressure surpassing 90 mm Hg. Hypertension can strain the heart, leading to cardiac issues, strokes, kidney complications, and other severe conditions [4]. Consequently, the prevention and management of the “three highs” are imperative for maintaining optimal health and diminishing the risk of cardiovascular disease. Starting from the dual perspectives of market and safety, utilizing food-derived compounds for the prevention and treatment of chronic inflammation is a strategy worthy of promotion.

**Figure 1:**
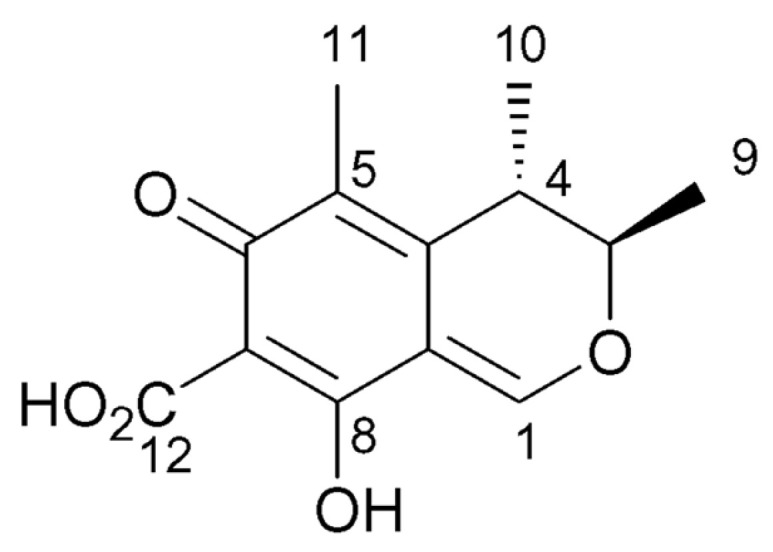
Chemical structure of citrinin.

*Monascus*, with a rich historical background, holds widespread applications in both culinary practices and traditional medicine [5,6]. Its bioactive components confer distinctive flavors to food items and exhibit a spectrum of biological activities, including the regulation of blood lipids, blood sugar, and blood pressure, as well as antioxidant and antitumor properties [7]. These effects predominantly arise from bioactive compounds present in Monascus’ metabolites, such as Monacolin K (MK), γ-aminobutyric acid (GABA), *Monascus* pigment, etc. [8]. GABA can relax vascular smooth muscle, reduce blood pressure [9], improve the function of β cells, promote insulin secretion, and reduce blood sugar levels by regulating apoptosis and the proliferation of β cells and inducing the transformation of α cells into β cells [10]. MK, a form of lovastatin, acts as a potent 3-hydroxy-3-methylglutaryl coenzyme-A (HMG-CoA) reductase inhibitor, leading to decreased circulating cholesterol and triglyceride levels and a reduction in lipid concentrations [11,12]. Nevertheless, certain *Monascus* strain metabolites contain citrinin (CIT), the chemical structure of which is shown in Figure 1. CIT is a polyketide mycotoxin produced by fungi of the genera Aspergillus, Penicillium, and *Monascus*, often found in products fermented by these microorganisms from animal and plant sources [13]. It is primarily metabolized by the liver in the body, which can lead to liver cell damage, cause hepatitis, and may even result in kidney dysfunction or failure, as well as potential teratogenic and carcinogenic effects [14]. Therefore, appropriate treatment and the strict inspection of foods and their production materials, especially those fermented by Aspergillus, Penicillium, and *Monascus*, can prevent the intake of citrinin. This study also includes the detection and screening of citrinin in the metabolic products of *Monascus* to ensure the acquisition of safe *Monascus* strains, which is crucial for the production of safe foods utilizing these strains.

Therefore, the identification of *Monascus* strains possessing high efficacy while devoid of CIT is crucial for the production of *Monascus*-based products. This meticulous screening process ensures the safety and efficacy of *Monascus*-fermented foods, thereby fostering advancements in the health food industry. *Monascus*, a microorganism widely used in the fermentation of traditional Chinese foods, produces natural products that effectively lower blood sugar, blood lipids, and blood pressure, offering a safer alternative with fewer side effects compared to chemical drugs. Since high blood lipids, high blood sugar, and high blood pressure are often interrelated and collectively constitute the main risk factors for cardiovascular diseases, natural products that can simultaneously reduce all three have significant importance for public health. Despite the potential of *Monascus* to lower the “three highs”, the variety and content of natural products produced by different strains due to genetic differences or cultivation conditions vary, leading to differences in activity among strains. Additionally, *Monascus* can produce the harmful substance Citrinin during the fermentation process, as seen in the incident involving Kobayashi Pharmaceutical, where the excessive presence of Citrinin in *Monascus* products poses a potential threat to public health [15]. Therefore, this study aims to screen for *Monascus* strains that do not produce Citrinin and have significant potential to lower the “three highs”. This research not only helps to enhance the safety of *Monascus* products but also provides a natural and effective solution for lowering the “three highs”, offering insights into the use of natural fungi to produce healthy foods that address chronic diseases in humans.

The EWM-TOPSIS is a multi-criteria decision analysis method that integrates the entropy weight method (EWM) and the Technique for Order Preference based on Similarity to the Ideal Solution (TOPSIS) [16]. Compared to subjective weighting methods (such as the Analytic Hierarchy Process), EWM determines weights by calculating the entropy value of indicators, reducing subjective judgment and ensuring the objectivity of the screening process. TOPSIS provides a clear ranking by comparing the distance of alternative solutions to the ideal solution. The EWM-TOPSIS model is suitable for screening *Monascus* strains with multiple biological activities because it can comprehensively consider various evaluation indicators, such as the effects of lowering blood lipids, blood sugar, and blood pressure, as well as the safety of the strains. It objectively assigns weights to safety indicators, efficiently handles a large amount of data, provides a quantitative ranking, and scientifically screens the best strains [17,18]. Therefore, EWM-TOPSIS is an ideal tool for screening safe and effective *Monascus* strains in this study, ensuring that the selected strains are both safe and effective.

Therefore, this study aims to isolate, purify, and identify red yeast enzymes from traditional *Monascus* products that are free of citrinin and have strong comprehensive abilities to lower blood sugar, blood lipids, and blood pressure. It aims to provide a microbial source for the fermentation production of safe and effective foods that reduce blood lipids, blood sugar, and blood pressure. Additionally, it offers a reference and conceptual framework for using natural fungi to produce healthy foods that address chronic diseases in humans.

## 2. Materials and Methods

### 2.1. Reagents and Materials

#### 2.1.1. *Monascus* Isolation Material

A total of 16 batches of *Monascus*-fermented food were selected from Guangxi, Guangdong, Fujian, and other places in China, including 10 parts Hongqu glutinous rice wine grains (HGG), 2 parts *Monascus* powder, 1 part red vinasse acid, 1 part Hongqu sufu, 1 part Red Kojic Rice, 1 part *Monascus* rice wine, and outdoor natural substrate fermentation samples.

#### 2.1.2. Preparation of Metabolites from *Monascus* Strains

Firstly, the preserved *Monascus* strains were activated in the Potato Dextrose Agar (PDA) medium. After activation, the *Monascus* mycelia were inoculated into the Potato Dextrose Broth (PDB) medium and placed in a constant temperature shaker at 28 °C and 160 rpm until the liquid medium turned orange-red and the mycelium balls were uniform, thus obtaining the fermented seed liquid. The bacterial concentration of the *Monascus*-fermented seed liquid was calculated using the plate colony counting method uniformly adjusted to 5 × 10^5^ cfu/mL. One milliliter of each bacterial solution was then transferred into conical flasks containing 150 mL of PDB culture medium and cultured in a constant temperature shaker at 28 °C and 160 rpm for 7 days to prepare the fermented stock solution, which was used to study the enzyme activities related to the secondary metabolites produced by *Monascus*.

#### 2.1.3. Reagents, Preparation of Standards, and Sample

(1) Acarbose and MK were acquired from Shanghai Aladdin Biochemical Technology Co., Ltd. (Shanghai, China). 4-Nitrophenyl-α-D glucopyranoside (pNPG), GABA, Fast Blue B zinc salt, and citrinin were purchased from Shanghai Maclin Biochemical Technology Co., Ltd. (Shanghai, China). Captopril, orlistat, pancreatic lipase, α-glucosidase (derived from yeast), and α-amylase (derived from pancreatic lipase) were purchased from Shanghai Yuanye Biotechnology Co., Ltd. (Shanghai, China). The angiotensin-converting enzyme was purchased from Shanghai Yingxin Laboratory Equipment Co., Ltd. (Shanghai, China). Acetonitrile (HPLC grade) and methanol (HPLC grade) were purchased from Thermo Fisher Technology Co., Ltd. (Shanghai, China). iMark was purchased from Bio-Rad (Waitsfield, VT, USA) and the High-Performance Liquid Chromatograph (E2695) was purchased from Waters Technology Co., Ltd. (Shanghai, China). The electron microscope (DM3000 LED) was purchased from Leica Microsystems (Wetzlar, Germany).

(2) MK and CIT reference substances were precisely weighed at 1.0 mg each and dissolved in 10 mL of chromatographic-grade methanol. Similarly, the GABA reference material, also weighing 1.0 mg, was accurately measured and dissolved in 10 mL of deionized water to create a single standard reserve solution, which was subsequently stored at 4 °C. The standard stock solution was then appropriately diluted with a solvent to generate standard solutions with concentrations of 500 μg/mL, 250 μg/mL, 125 μg/mL, 62.5 μg/mL, and 31.25 μg/mL. Before utilization, the standard solution underwent filtration through a 0.22 μm microporous membrane and was employed to construct standard curves for MK, CIT, and GABA.

(3) Preparation of *Monascus purpureus* liquid extracts (MPLs). Following the completion of *Monascus* liquid fermentation, the liquid underwent treatment using a high-speed disperser (XHF-DY, Ningbo Xinzhi Biotechnology Co., Ltd., Ningbo, China) at 10,000 rpm for 0.4 min. Subsequently, the liquid was filtered, and the filtrate was concentrated under reduced pressure and quantified. The resulting extract was then formulated into MPLs with a concentration of 3 mg/mL and preserved at 4 °C. These MPLs were utilized to assess pancreatic lipase inhibitory activity, α-glucosidase inhibitory activity, α-amylase inhibitory activity, angiotensin-converting enzyme inhibitory activity, and GABA content determination.

The triploid methanol solution was introduced into the *Monascus* fermentation broth and processed following the aforementioned procedure. Subsequently, MPLs were extracted using alcohol with a concentration of 3 mg/mL and were preserved at 4 °C. These MPLs were utilized to analyze the content of MK and CIT.

### 2.2. Methods

#### 2.2.1. Isolation, Purification, and Identification of *Monascus*

The collected *Monascus* products were treated using the following three methods: (1) *Monascus* products were transferred into aseptic centrifuge tubes under sterile conditions, and aseptic water was added to achieve a total volume of 10 mL. (2) In a sterile environment, a small portion of the *Monascus* products was uniformly selected and placed on a PDA medium. (3) Under aseptic conditions, a small quantity of *Monascus* products was placed in a sterile centrifuge tube, and 10 mL of sterile water was added. This mixture was thoroughly mixed and then diluted into six gradients. From these dilutions, 100 μL was pipetted and evenly spread on the surface of the PDA medium. The culture was then incubated at 28 °C until the red hyphae resembling *Monascus* were visible to the naked eye. The visible hyphae were collected using an inoculation ring and transferred to a PDA medium, where the inoculum germinated and formed colonies. These colonies, consistent in color and shape due to repeated isolation and purification on the same plate, were subsequently stored at 4 °C.

(1) Method of colony morphology identification. The selected *Monascus* strains were inoculated onto PDA medium using a three-point inoculation method and incubated at 28 °C for 7 days. After incubation, the characteristics of the colonies were observed and recorded. These characteristics included colony size, color, edge morphology, and other relevant features.
(2) Identification of microscopic morphology. The microscopic morphological characteristics of the hypha, conidia, and chasmothecium of *Monascus* were observed and documented through film observation. Refer to [19] for the preliminary identification of *Monascus* based on colonial morphology and individual microscopic morphologies.
(3) Molecular biological identification. Universal primers ITS1 and ITS4 were used as primers for the PCR test, and a BLAST comparison was conducted following sequencing.

#### 2.2.2. Determination of Pancreatic Lipase (PL) Inhibitory Capacity

The PL inhibitory activity of MPLs was determined concerning the method [20] and slightly modified to evaluate the lipid-lowering activity of the identified metabolites from *Monascus* metabolites. The 0.05 M Tris-HCl buffer at pH 7.0, 1 mg/mL of the alpha-naphthyl acetate ester, and 2 mg/mL of the fatty enzyme solution were added to each MPL with a certain concentration gradient, mixed well, and incubated at 37 °C for 20 min before 1 mg/mL Fast Blue B zinc salt was added. The light absorption value was measured at 520 nm, and the inhibition rate of PL was calculated. The positive control was orlistat. In the control group, a buffer solution was used instead of an enzyme solution, and the inhibition rate was calculated according to Formula (1):

(1) $$\mathrm{Inhibition}(\%)=1-\;(\frac{B\;-\;b}{A\;-\;a})\;\times \;100\%$$

In the formula, A is the absorption value of the blank group; a is the absorption value of the blank control group; B is the absorption value of the sample test group; and b is the absorption value of the sample control group.

#### 2.2.3. Determination of α-Glucosidase (α-Glu) Inhibitory Capacity

The α-Glu inhibitory activity of MPLs was determined by the pNPG method, and an evaluation of the blood-glucose-lowering ability of *Monascus* metabolites was conducted. PBS and α-Glu were preheated at 37 °C for 5 min in advance, and 100 μL of PBS (pH 6.8, 0.1 M), 20 μL of α-Glu and 20 μL of the sample test solution with different concentration gradients were added successively, mixed well, and reacted at 37 °C for 15 min; then, 20 μL of 8 mM pNPG was added, and then mixed and reacted at 37 °C for 15 min, and 140 μL of 1M Na_2_CO_3_ was added to terminate the reaction, and α-Glu was replaced by 20 μL PBS as the sample control group. Distilled water was used as the empty white group instead of the liquid to be measured, and acarbose was used as the positive control. The light absorption value was determined to be 405 nm. The α-Glu inhibitory activity of the sample was calculated according to Formula (1).

#### 2.2.4. Determination of α-Amylase (α-Amy) Inhibitory Capacity

The α-Amy inhibitory activity of MPLs was determined by iodine colorimetry [21], and the hypoglycemic ability of *Monascus* metabolites was evaluated. PBS and α-Amy were preheated at 37 °C for 5 min in advance; then, 80 μL of PBS (pH 6.8, 0.1 M), 20 μL of α-Amy and 40 μL of MPLs with different concentration gradients were added successively, mixed, and reacted at 37 °C for 15 min. We added 20 μL of 0.5% starch, mixed well, and watched it react at 37 °C for 15 min before adding 20 μL of 1M H_2_SO_4_ to terminate the reaction, and finally adding 20 μL of 4 mM iodine solution, replacing α-Amy with 20 μL of PBS as the sample control group, and replacing the test solution with distilled water as the blank group. Acarbose was used as a positive control, and the absorbance value was determined at 660 nm. The α-Amy inhibitory activity of the sample was calculated according to Formula (1).

#### 2.2.5. Determination of Angiotensin-Converting Enzyme (ACE) Inhibitory Capacity

The ACE inhibitory activity of MPLs in vitro was determined by referring to Sofia’s method [22] with some modifications. The test sample was added at 40 μL, 0.1 U/mL of the ACE test agent was added at 10 μL, 1 mmol/L of the FAPGG substrate was added at 50 μL, and the control group was replaced with a 40 μL HEPES buffer (80 mmol/L). The absorbance at the 340 nm wavelength was measured immediately after the addition of the reagent, but the absorbance at the 340 nm wavelength was measured again immediately after the reaction at 37 °C for 30 min. ACE inhibitory activity was calculated according to Formula (1).

#### 2.2.6. Determination of CIT Content

(1) Drawing the standard curve: The standard series of the CIT working liquid prepared in Section 2.1.3 was filtered into the brown liquid phase bottle with an organic filtration membrane of 0.22 μm. The standard curve of CIT was drawn with the series concentration of the CIT standard solution as the horizontal coordinate and the chromatographic peak area as the vertical coordinate, and the linear regression equation and correlation coefficient were obtained.

(2) High-performance liquid chromatography (HPLC) is used for the detection of the CIT: JADE-PAK ODS C18 column (250 × 4.6 mm, 5 µm). The column temperature was 28 °C, and the mobile phase was V (acetonitrile): V (2% acetic acid) = 46:54. The flow rate was 1 mL/min, the injection volume was 10 μL, and the detection wavelength was 330 nm.

#### 2.2.7. Determination of MK Content

The MK standard curve was drawn in the same way as Section 2.2.6 HPLC detection conditions as follows: JADE-PAK ODS C18 column (250 × 4.6 mm, 5 µm). The column temperature was 28 °C, and the mobile phase was V(acetonitrile): V(0.5% phosphoric acid) = 60:40. The flow rate was 1 mL/min, the injection volume was 10 μL, and the detection wavelength was 237 nm.

#### 2.2.8. Determination of GABA Content

The GABA standard and samples to be tested were derivatized according to the method described in reference [23]. Sequentially, 30 μL of the sample solution was added with 30 μL of 0.4 mol/L potassium borate buffer (pH 8.6), followed by 60 μL of 5 mmol/L FMOC-Cl, and the mixture was reacted at 25 °C for 20 min. Then, 30 μL of 1 mol/L glycine and 30 μL of 0.1% acetic acid solution were added, followed by dilution with 120 μL of distilled water. The mixture was filtered through a 0.22 μm nylon filter membrane, and the filtrate was stored in a 4 °C refrigerator for subsequent testing. The drawing of the GABA standard curve is the same as Section 2.2.6. The detection conditions were as follows: the JADE-PAK ODS C18 column (250 × 4.6 mm, 5 µm) was used, the volume ratio of acetonitrile to 0.1% acetic acid in the mobile phase was 55:45, the column temperature was 30 °C, the flow rate was 1 mL/min, the injection volume was 10 μL, and the detection wavelength was 254 nm.

#### 2.2.9. Comprehensive Evaluation of *Monascus* Strains by EWM-TOPSIS

Traditional evaluation methods often rely on a single index or simple statistical analysis, and it is difficult to reflect the comprehensive effect of *Monascus* strains. This study introduces a systematic and scientific evaluation method to screen a safe *Monascus* strain with activity reducing the three highs based on the CIT content and multiple inhibition rates.

EWM is an objective weighting method that determines the weights of evaluation indicators based on the concept of information entropy. The EWM is widely used in the field of multi-attribute decision analysis and comprehensive evaluation. The basic principle of the entropy weight method is to determine the weights of evaluation indicators based on the concept of information entropy [24]. In this study, the EWM was used to calculate the weight relationship between the inhibition rate indexes, which provided the basis for the subsequent multi-index comprehensive evaluation of *Monascus* with activity to reduce the three highs (hyperlipidemia, hyperglycemia, and hypertension).

(1) Construct a standardized matrix.

Below, the number of rows represents the number of evaluation objects, and the number of columns represents the number of evaluation criteria to determine the specific quantitative value of each index of each *Monascus* strain and form the original data matrix:

(2) $$\;X_{\mathrm{mn}}=\left[\begin{array}{c} \begin{array}{ccc} X_{11} & X_{12} & \ldots \\ X_{21} & X_{22} & \ldots \\ \vdots & \vdots & \ddots \end{array}\\ \begin{array}{ccc} X_{m1} & X_{m2} & \ldots \end{array} \end{array}\begin{array}{c} X_{1n}\\ X_{2n}\\ \begin{array}{c} \vdots \\ X_{mn} \end{array} \end{array}\right]$$

where X represents the original data matrix, and X_mn_ is the evaluation index value for the nth enzyme activity of the mth *Monascus* strain.

(2) Data standardization processing.

Since indexes of different properties have a greater impact on the results, they should be normalized into dimensionless matrices to facilitate a comparison between indexes. The processing process for positive indicators is shown as follows:

(3) $$b_{\mathrm{ij}}=\frac{X_{ij}-min(X_{j})}{max(X_{j})-min(X_{j})}$$

To ensure that the values are valid, an effective value of 0.0001 is added to each value after being dimensionless. x_ij_ indicates the ratio of candidate strains to the evaluation criteria. Here, max x_j_ represents the optional maximum value of evaluation criterion j, and min x_j_ represents the optional minimum value of evaluation criterion j.

(3) Define standardized values.

The proportion of the nth evaluation criterion of each evaluation object is calculated according to Formula (4).

(4) $$P_{\mathrm{ij}}=\frac{b_{ij}}{\sum_{i=1}^{m}y_{ij}}$$

where P_ij_ is the contribution value of the ith evaluation object selected under the j evaluation criterion under the normalized initial matrix.

(4) Entropy determination.

Furthermore, the entropy of each evaluation criterion was calculated according to Formula (5).

(5) $$e_{j}=-\frac{1}{\ln n}\sum_{i=1}^{n}p_{ij}\ln p_{ij}$$

(5) The degree of variation in the evaluation criteria was calculated.

The variation degree of evaluation criterion j was calculated according to Formula (6). Its meaning is the degree of fluctuation of the contribution value of the alternative under the j criterion.

(6) $$g_{j}=1-e_{j}$$

(6) Calculate the weight of the evaluation criteria.

The weight Wj of the evaluation criterion of item j is calculated according to Formula (7).

(7) $$W_{j}=\frac{g_{j}}{\sum_{i=1}^{m}g_{j}}$$

TOPSIS is a multi-attribute decision-making method used to select the best solution from a set of alternatives [25]. The core idea of the TOPSIS method is to evaluate and select schemes based on the relative proximity to positive and negative ideal solutions and to consider the weights and preferences of all attributes, providing a systematic decision-making framework for decision-makers [26].

The TOPSIS method involves constructing an initial matrix, which is normalized to eliminate the impact of different dimensions, determining index weights, conducting a comprehensive evaluation of *Monascus* strains, obtaining positive and negative ideal solutions, calculating their relative closeness based on the distance to ideal solutions, and determining the optimal *Monascus* strain. The specific steps are shown as follows:

(7) Determine the weighted normalized matrix.

Weight is allocated according to the relative importance of each evaluation criterion. The main purpose of this step is to reduce the influence of subjective factors. The weight calculated in the previous step is multiplied by standardized data, as shown below:

(8) $$Z_{ij}=b_{ij}w_{j}$$

(8) Determine ideal positive and negative measures.

Formulas (9) and (10) are used to determine the best and worst measurements from the weighted normalized matrix. The purpose of this calculation step is to define different indicators. The closer the vector value of the indicator is to the positive ideal solution, the better the performance and the worse the performance is.

(9) $$Z_{j}^{+}=max\left(Z_{1j},Z_{2j},\cdots Z_{nj}\right)$$

(10) $$Z_{j}^{-}=min\left(Z_{1j},Z_{2j},\cdots Z_{nj}\right)$$

(9) Identification of Euclidean distance.

The Euclidean distance is obtained from the positive and negative ideal solutions to measure the similarity of each evaluation object to the ideal solution. The calculation formula is as follows:

(11) $$d_{i}^{+}=\sqrt{\sum_{j=1}^{n}\left(c_{ij}-c_{j}^{+}\right)}\wedge 2\;\;\;\;d_{i}^{-}=\sqrt{\sum_{j=1}^{n}\left(c_{ij}-c_{j}^{-}\right)}\wedge 2$$

(10) Relative closeness and ranking of alternatives.

The higher the degree of proximity, the higher the ranking, indicating that the overall evaluation criteria of the evaluation object showed a stronger potential.

(12) $$c_{i}=\frac{Z_{j}^{-}}{Z_{j}^{+}+Z_{j}^{-}}$$

#### 2.2.10. Statistical Analysis

All experiments were conducted with three replicates, and the results were presented as the mean ± standard deviation (SD). Data processing and plotting were performed using MEGA-X64, SPSS Statistics 27, Origin 2018, Excel, and GraphPad Prism 8.0. In all statistical tests, significance levels were denoted as follows: * *p* < 0.05 signified a significant difference, and ** *p* < 0.01 indicated an extremely significant difference.

## 3. Results

### 3.1. Isolation of Monascus

The separation and purification of *Monascus* from *Monascus*-fermented food is not only the basic work of screening efficient functional strains but also a key step to ensure the quality and safety of *Monascus*-fermented food. Following the separation and purification process, fourteen mold strains were successfully isolated, exhibiting an appearance resembling *Monascus* (initially white colonies transitioning to red or orange pigmentation over time) from the acquired *Monascus* products. These isolated strains were designated as M1 to M14. The details of the *Monascus* isolation process are presented in Table 1.

### 3.2. Identification of Monascus

The identification of isolated and purified *Monascus* strains is a critical step in ensuring the accuracy of the study and the safety of the fermentation products. The identification results serve as foundational data for further research on the functionality of these strains and play a decisive role in the study’s objective of identifying efficient and safe *Monascus* strains.

**Figure 2:**
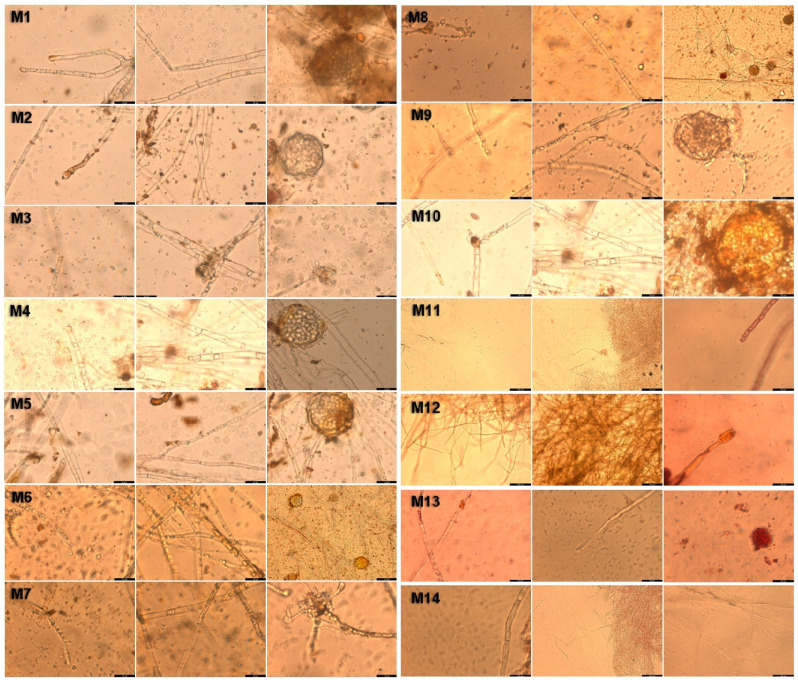
Microscopic characteristics of isolated strains. (20 μm).

After separation and purification, the 14 strains obtained were inoculated on a PDA medium and incubated at 28 °C for 7 days. The culture characteristics of each strain are shown in Table 2, and the specific morphological characteristics are described in Table 3. To further observe the microscopic morphology of these strains, a new hypha from the outside of the colony was made, and the microscopic morphology was observed. According to the observation results (Figure 2), the microscopic morphology of M1 to M10 strains was consistent with the typical microscopic morphological characteristics of *Monascus*. The conidia of strains M1 to M10 grow at the tips of the hyphae, are rounded, and have a surface without attachments; the hyphae of strains M1 to M6 contain a small amount of content, have no attachments on the outer wall and possess septa. The hyphae of strains M7 to M10 do not contain content, have no attachments on the outer wall, and possess septa. The chasmothecium of strains M1, M2, M4, M5, M8, M9, and M10 are regular in shape, have thin walls, are round, and the spores within are not very distinct and do not contain pigment granules; the chasmothecium of strains M3 and M7 are irregular in shape, radiate, with an unclear outer wall, and contain a small number of spores inside; the chasmothecium of strain M6 is regular in shape, with thick walls, round in shape, and the spores within are distinct and contain pigment granules. However, the M11 to M13 strains did not show obvious conidia and chasmothecium, which did not conform to the micromorphological characteristics of basic *Monascus* [27], so these strains were preliminarily excluded. The experimental results provide a reference for the subsequent identification of *Monascus* strains.

### 3.3. Molecular Identification of Monascus

Similarity analysis of sequences from BLAST and GeneBank databases provided by NCBI show that 10 strains were identified as *Monascus purpureus*. One strain was *Lecanicillium dimorphum*, one strain was *Fusarium kyushuense*; one strain was *Epicoccum dendrobii*; and one was *Forliomyces uniseptata*.

**Figure 3:**
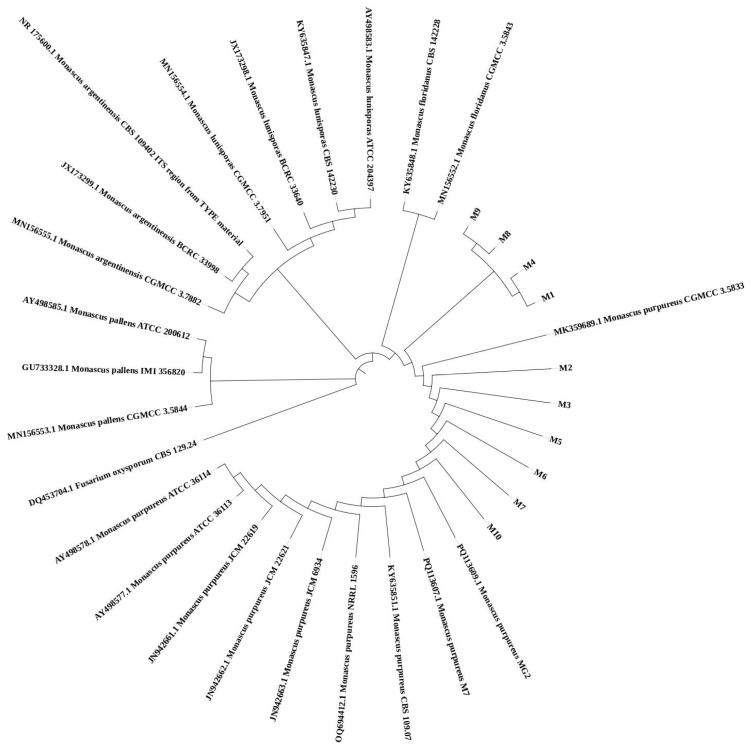
Phylogenetic tree of *Monascus purpureus* constructed using MEGA 7.0 software.

The BLAST program was employed to compare the sequence homology of the ITS gene sequences from ten strains of *Monascus purpureus*, and strains showing high sequence similarity were chosen. A phylogenetic tree was constructed using the Neighbor-Joining (NJ) method in MEGA 5.2.0 software, and the branch reliability of the evolutionary tree was assessed using node numbers. This approach facilitated the further determination of the strains, as illustrated in Figure 3.

The strains used in this study were M1-M10. The tree is rooted with *Fusarium oxysporum*.

### 3.4. Determination of Pancreatic Lipase (PL) Inhibitory Activity in MPLs

In the context of hyperlipidemia treatment, a prominent manifestation of metabolic disorders, PL stands out as a crucial target enzyme. Primarily responsible for breaking down triacylglycerol in the intestine into fatty acids and glycerol, PL facilitates the absorption of these decomposition products into the bloodstream via the small intestine, thereby influencing lipid levels [28]. This study aimed to assess the PL inhibitory potential of 10 *Monascus* strains to identify those with promising hypolipidemic properties.

**Figure 4:**
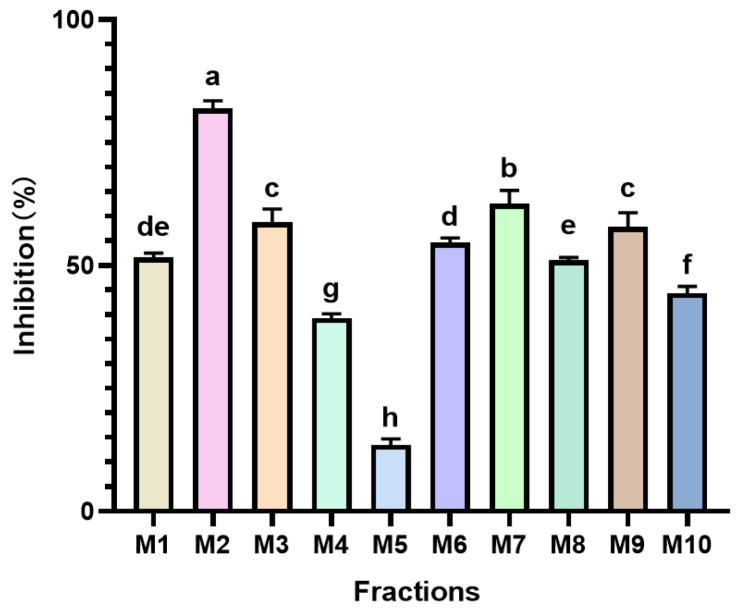
Inhibitory activity of different MPLs on PL. Values are the mean of three inhibition rates (*n* = 3), with error bars representing the standard deviation (SD) of the mean values. Different lower-case letters represent significant differences (*p* < 0.05) between different samples.

The findings are depicted in Figure 4 and revealed that all 10 *Monascus* strains exhibited notable PL inhibitory activities, indicating the potential of *Monascus* in modulating blood lipids. Among these strains, the M2 *Monascus* strain demonstrated the most robust PL inhibitory activity, with an impressive inhibition rate of 81.903%, significantly distinguishing it from the other strains (*p* < 0.05). This outcome suggests that the M2 strain may harbor a higher concentration of lipase inhibitors or possess a distinctive metabolic pathway capable of effectively suppressing PL activity, thereby curtailing fat absorption and aiding in lipid reduction. Conversely, the M5 strain displayed a relatively lower PL inhibitory activity (13.463%), which is a characteristic that could be attributed to its genetic profile or metabolite variations. Differences in the genetic background among different *Monascus* strains may lead to variations in the types and quantities of their metabolic products, thereby affecting their biological activities. The PL inhibitory activities of the remaining strains ranged from 39.256% to 62.500%, signifying their potential for lipid reduction, albeit being generally inferior to the M2 strain.

**Figure 5:**
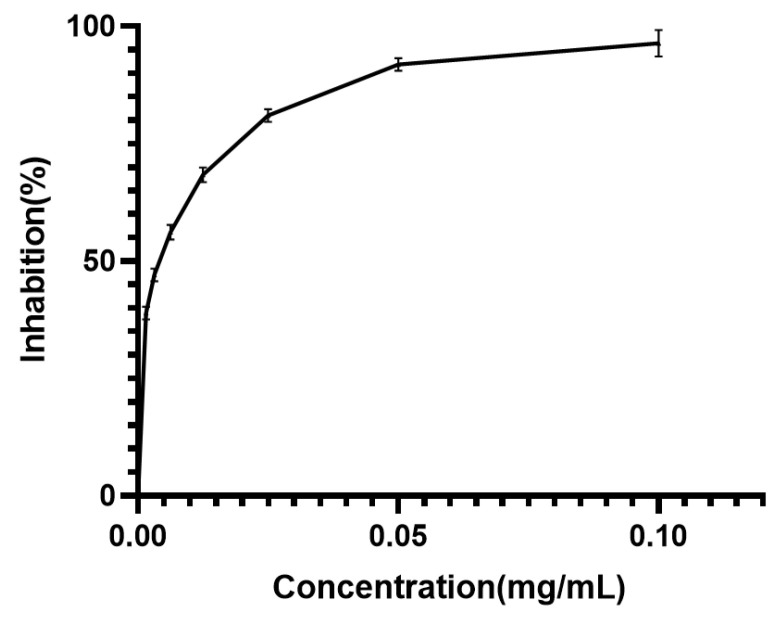
Inhibitory activity of orlistat on PL. Values are the mean of three inhibition rates (n = 3), with error bars representing the standard deviation (SD) of the mean values.

To provide a benchmark, the PL inhibitory activity of orlistat, a recognized positive control, was also examined. The results illustrated in Figure 5 indicate that the PL inhibitory activities of all MPLs were lower than those of orlistat. While *Monascus* exhibited considerable PL inhibitory effects, its efficacy might not match that of commercial weight loss medications. Nevertheless, *Monascus*, as a naturally sourced microorganism, offers higher safety levels, rendering it a more favorable candidate as a functional food additive for hyperlipidemia management.

### 3.5. Determination of α-Glucosidase (α-Glu) Inhibitory Activity in MPLs

In the realm of diabetes treatment and prevention strategies, α-Glu emerges as a pivotal therapeutic target. Situated at the brush border of the small intestinal mucosa, this enzyme plays a crucial role in breaking down complex carbohydrates from food into monosaccharides, facilitating sugar absorption by the intestines [29]. By impeding α-Glu activity, it is feasible to decelerate carbohydrate breakdown, diminish glucose absorption in the intestine, lower blood sugar levels, and elevate water content [30].

**Figure 6:**
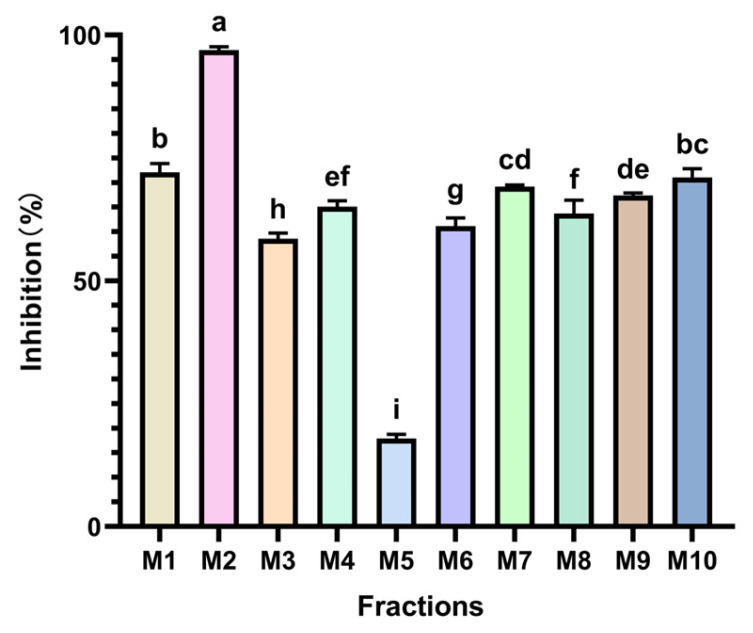
Inhibitory activity of different MPLs on α-Glu. Values are the mean of three inhibition rates (n = 3), with error bars representing the standard deviation (SD) of the mean values. Different lower-case letters represent significant differences (*p* < 0.05) between different samples.

This study aimed to assess the hypoglycemic potential of all MPLs through their ability to inhibit α-Glu. The experimental outcomes are presented in Figure 6, highlighting the significant α-Glu inhibitory activity of the M2 strain, which exhibited an impressive inhibition rate of 96.938%, markedly surpassing that of other strains (*p* < 0.05). This finding suggests that the M2 strain may harbor potent α-Glu inhibitors, indicating promising hypoglycemic effects. Conversely, the α-Glu inhibitory activity of the M5 strain was modest, standing at only 17.937%, underscoring the variability in bioactive constituents among different *Monascus* strains. The activities of reducing blood lipids, blood sugar, and blood pressure are usually associated with the secondary metabolic products of the strains. If the secondary metabolic pathways of certain strains are blocked, it may reduce the production of these active compounds. The α-Glu inhibitory activity of the remaining *Monascus* strains ranged from 58.540% to 73.080%, indicating their potential for blood sugar reduction, albeit generally lower than the M2 strains.

**Figure 7:**
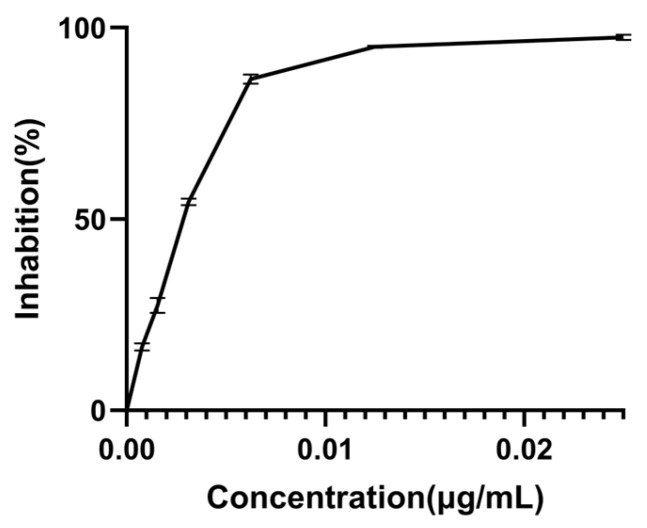
Inhibitory activity of acarbose on α-Glu. Values are the mean of three inhibition rates (n = 3), with error bars representing the standard deviation (SD) of the mean values.

It is noteworthy that the α-Glu inhibitory activity of all *Monascus* strains was inferior to that of the positive control acarbose, a recognized α-Glu inhibitor utilized in type 2 diabetes management [31] (Figure 7). This observation suggests that while *Monascus* exhibits hypoglycemic potential, its efficacy may not be adequate to supplant current anti-diabetic medications.

### 3.6. Determination of α-Amylase (α-Amy) Inhibitory Activity in MPLs

In the realm of blood sugar regulation, α-Amy plays a crucial role by breaking down starch into smaller sugar molecules like maltose, which are further converted into glucose by α-Amy in the small intestine. This glucose is subsequently absorbed into the bloodstream, leading to an increase in blood sugar levels. By modulating α-Amy activity, the rate of starch breakdown can be effectively managed, indirectly controlling the speed and magnitude of blood sugar elevation. This strategy holds significant importance in the management of blood sugar levels for diabetic patients [32,33].

**Figure 8:**
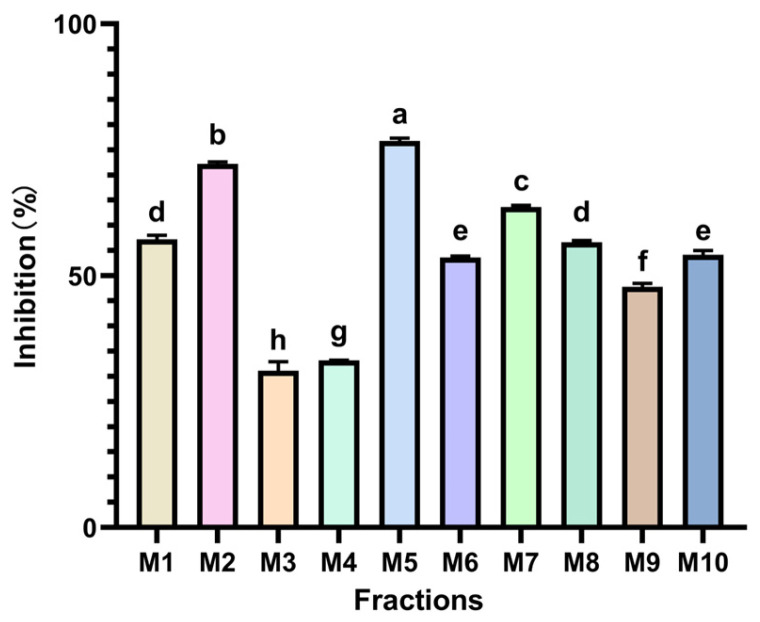
Inhibitory activity of different MPLs on α-Amy. Values are the mean of three inhibition rates (n = 3), with error bars representing the standard deviation (SD) of the mean values. Different lower-case letters represent significant differences (*p* < 0.05) between different samples.

**Figure 9:**
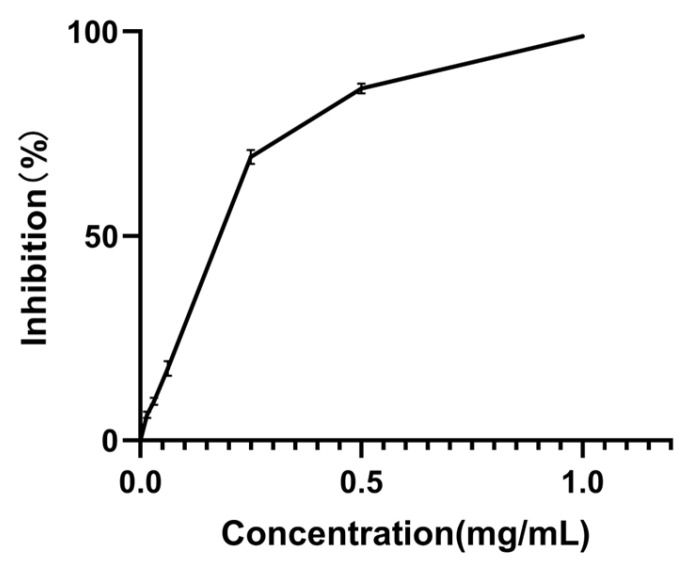
Inhibitory activity of acarbose on α-Amy. Values are the mean of three inhibition rates (n = 3), with error bars representing the standard deviation (SD) of the mean values.

The findings of this study, depicted in Figure 8, reveal notable variations in α-Amy inhibitory activities among the ten *Monascus* strains. In particular, the M5 strain exhibited remarkable α-Amy inhibitory effects, with inhibition rates of 76.733% and 72.215%, respectively, displaying significant disparities compared to the other strains (*p* < 0.05). The pronounced activities observed in the M5 and M2 strains may be attributed to the presence of distinct active ingredients that possess α-Amy inhibitory properties. The α-Amy inhibitory activities of the remaining *Monascus* strains ranged from 31.144% to 63.650%, falling below those of the positive control, acarbose, as illustrated in Figure 9.

### 3.7. Determination of Angiotensin-Converting Enzyme (ACE) Inhibitory Activity in MPLs

In cardiovascular physiology, the renin–angiotensin system and the kallikrein–kinin system stand as pivotal regulators of blood pressure and fluid equilibrium. Within these systems, ACE assumes a crucial role as a converting enzyme, catalyzing the transformation of inactive angiotensin I into angiotensin II, a potent vasoconstrictor, while also degrading bradykinin, a vasodilator [34]. Consequently, ACE inhibitors play a significant role in hypertension treatment by reducing angiotensin II production and elevating bradykinin levels [35].

**Figure 10:**
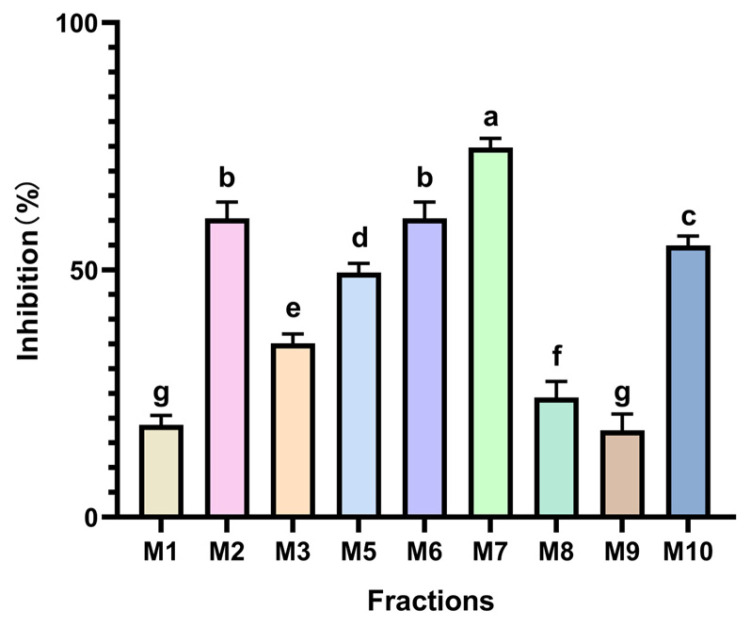
The inhibitory activity of different MPLs on ACE. Values are the mean of three inhibition rates (n = 3), with error bars representing the standard deviation (SD) of the mean values. Different lower-case letters represent significant differences (*p* < 0.05) between different samples.

The evaluation of the antihypertensive activity of MPLs was conducted through an ACE inhibition test, with the results depicted in Figure 10. Notably, the M7 strain exhibited the most robust inhibitory effect, with an inhibitory capacity of 74.725%, demonstrating a significant variance compared to other groups (*p* < 0.05). Conversely, the M4 strain displayed no ACE inhibitory activity, indicating variations in bioactive components among the different *Monascus* strains. This variance opens up possibilities for the screening and cultivation of strains with specific pharmacological effects.

**Figure 11:**
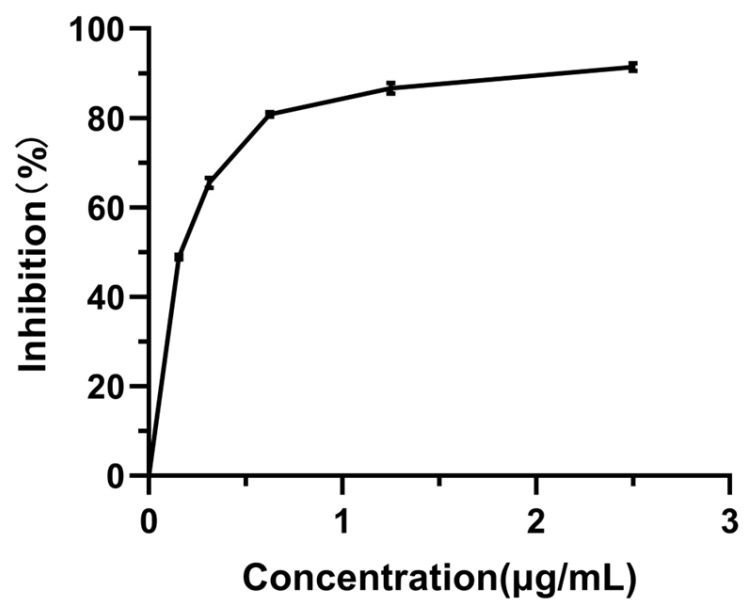
The inhibitory activity of acarbose on ACE. Values are the mean of three inhibition rates (n = 3), with error bars representing the standard deviation (SD) of the mean values.

While the ACE inhibitory activity of the remaining *Monascus* strains was lower than that of the positive control captopril (Figure 11), the activity range of 16.484% to 61.538% still holds biological relevance and suggests potential utilization as a food ingredient for hypertension prevention. ACE inhibitors sourced from natural origins may offer enhanced safety and tolerability compared to synthetic drugs, rendering them suitable for long-term consumption [36].

### 3.8. Analysis of CIT Content in MPLs

**Figure 12:**
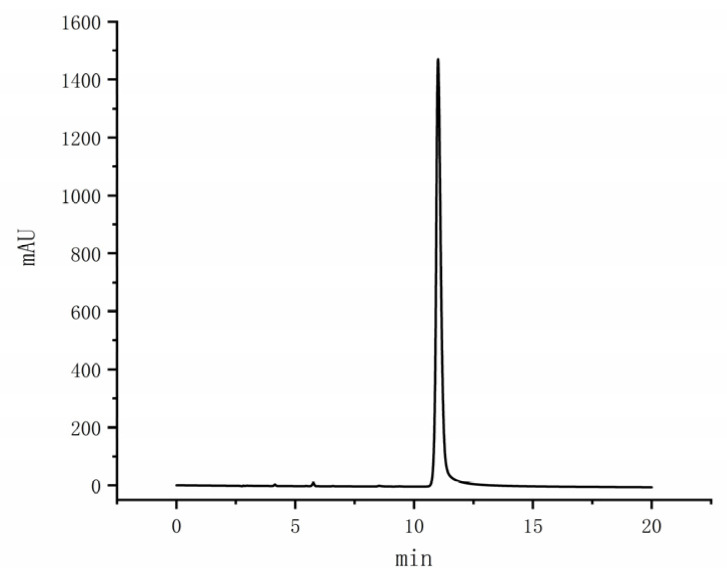
Liquid chromatogram of CIT (1 mg/mL).

CIT is a mycotoxin synthesized by a specific fungus, including certain species of *Monascus* [37]. The presence of CIT presents a potential health hazard, underscoring the importance of monitoring the hesperidin content in *Monascus* metabolites during screening and application processes. The standard curve for CIT was established following the Section 2.2.6 detection method, yielding the equation Y = 379.6X + 1.777, with R^2^ = 0.9985. Notably, the chromatographic analysis in Figure 12 of the CIT standard revealed a peak at 11.01 min with a well-defined peak shape. The limit of detection was determined to be 1.2307 ng/mL, while the limit of quantification stood at 5.0883 ng/mL.

**Figure 13:**
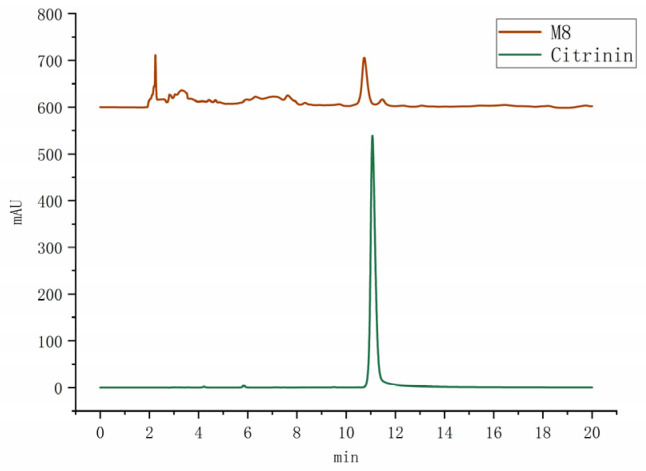
HPLC chromatogram of CIT in *Monascus* strain.

As illustrated in Figure 13, only the M8 strain exhibited the presence of CIT, with a content of 2.301 mg/g. This finding raises concerns regarding food safety and the potential health risks associated with the M8 strain, while the other nine *Monascus* strains were identified as safe varieties.

### 3.9. Analysis of MK Content in MPLs

MK is the key active ingredient in *Monascus*-fermented products, which exists in two forms: the acid type (open loop) and lactone type (closed loop) [38]. The acid type MK (MKA) showed superior HMG-CoA reductase inhibition compared to the lactone type MK (MKL) in regulating blood lipids and cholesterol [39]. HMG-CoA reductase is a key rate-limiting enzyme in the cholesterol biosynthesis pathway, and the inhibition of its activity directly affects the level of cholesterol synthesis [40]. MKA, due to its open-loop structure, can bind to the active site of HMG-CoA reductase more effectively, thus inhibiting the activity of the enzyme more effectively; therefore, it shows significant biological activity in lowering blood lipids and blood sugar.

**Figure 14:**
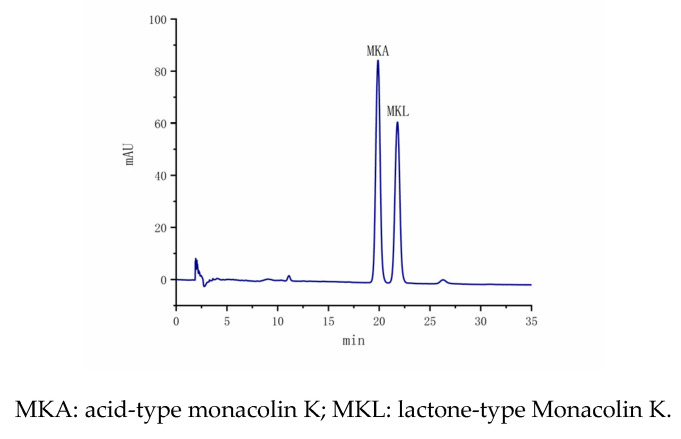
HPLC chromatogram of MK.

The MK standard curve was determined according to the Section 2.2.6 detection method, and the results are shown in Figure 14. MKA and MKL peaked at 19.12 min and 21.13 min, respectively, and the peak pattern was good. The standard curves were well fitted linearly; the MKA regression equation was Y = 0.4366X + 0.3920, R^2^ = 0.9991, and the MKL regression equation was Y = 0.3579X + 0.1647, R^2^ = 0.9992. The detection limit of the total MK content was 2.0918 mg/mL, and the quantification limit was 5.8411 mg/mL.

**Figure 15:**
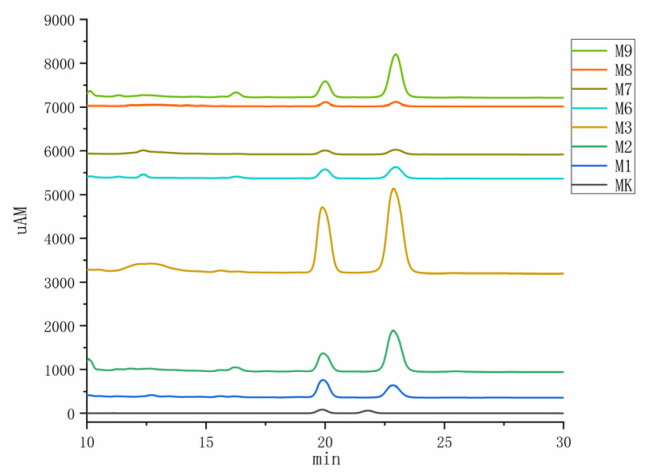
HPLC chromatogram of MK in *Monascus* strain.

**Figure 16:**
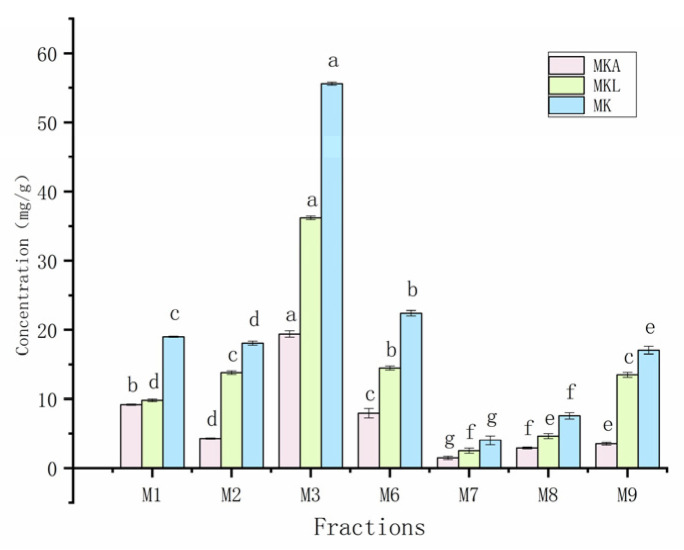
Content map of MK in the *Monascus* strain. Values are the mean of three inhibition rates (n = 3), with error bars representing the standard deviation (SD) of the mean values. Different lower-case letters represent significant differences (*p* < 0.05) between different samples.

As depicted in Figure 15, the metabolites of *Monascus* strains M1, M2, M3, M6, M7, M8, and M9 contained MK, albeit with variations in content levels. Notably, the extract from the M3 strain displayed the highest MK content (Figure 16), measuring 55.606 mg/g, and signifying the superior MK production capability of the M3 strain. The accurate determination of the MK content not only aids in the selection of *Monascus* strains with efficient MK production but also furnishes essential data supporting the application of *Monascus* in the realms of medicine and healthy food.

### 3.10. Analysis of GABA Content in MPLs

**Figure 17:**
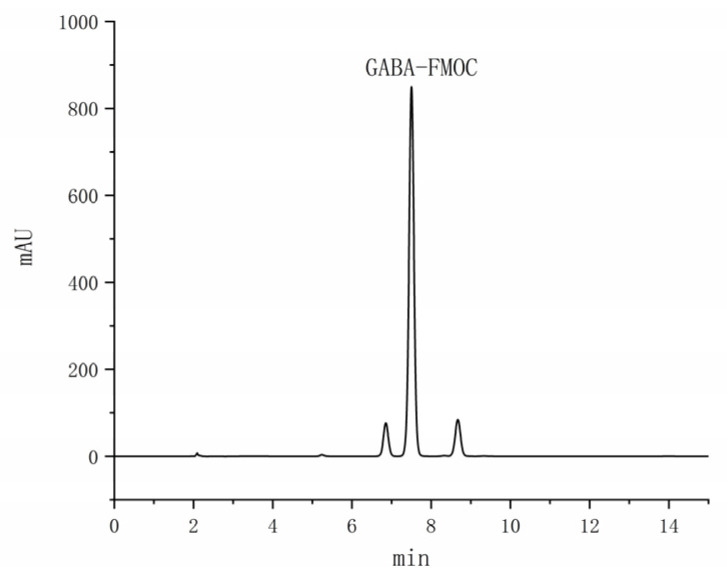
HPLC chromatogram of GABA.

GABA is a naturally occurring non-protein amino acid that holds significant physiological importance in living organisms, particularly in its roles in blood pressure reduction, blood sugar level modulation, and depression alleviation [41]. The standard curve for GABA, established through the 2.19 test square method, is represented by the equation Y = 27.00X + 0.9352, R^2^ = 0.9994. An examination of Figure 17 reveals GABA peaking at 7.49 min with a well-defined peak shape. The limit of detection was determined to be 0.0082 mg/mL, while the limit of quantification was 5.5620 mg/mL.

**Figure 18:**
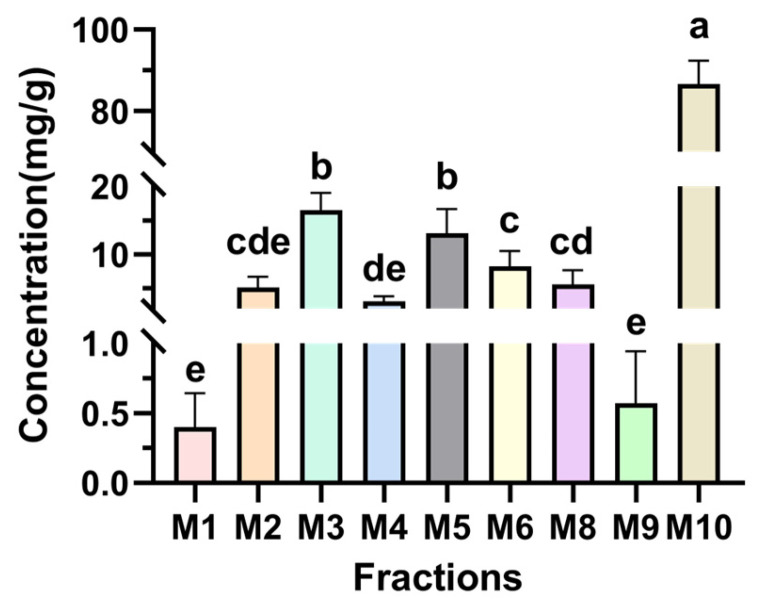
Content map of GABA in the *Monascus* strain. Values are the mean of three inhibition rates (n = 3), with error bars representing the standard deviation (SD) of the mean values. Different lower-case letters represent significant differences (*p* < 0.05) between different samples.

Quantitative HPLC analysis of the fermentation broth from 10 *Monascus* strains, as depicted in Figure 18, shows that all strains except M7 contained GABA. These findings indicate the general capacity of *Monascus* strains to produce GABA during the metabolic process, albeit with variations among strains.

### 3.11. A Comprehensive Evaluation of Monascus Based on the EWM-TOPSIS

It is necessary to screen out safe *Monascus* strains with a stronger comprehensive ability to decrease the “three highs”. Given the significant differences in the bioactivity of the 10 *Monascus* strains in reducing blood lipids, blood sugar, and blood pressure, this study aimed to adopt a more objective screening method to identify the strain with the optimal function of lowering the three high indicators. This study conducted toxic substance screening and bioactivity evaluation of several *Monascus* strains. After ensuring that the screened strains did not contain harmful CIT, nine safe *Monascus* strains were selected as candidate strains. Five inhibitory rates of hypolipidemic activity (PL inhibitory activity), hypoglycemic activity (α-Glu and α-Amy inhibitory activity), and hypertensive activity (ACE inhibitory activity) were used as the evaluation criteria. To objectively evaluate the importance of these evaluation criteria, the EWM was used to determine the weight of each evaluation criterion, and then the TOPSIS method was used to comprehensively rank the candidate strains to select the best *Monascus* strains.

#### 3.11.1. Construct the Decision Matrix

The original data matrix X (inhibition rate) was formed by the specific quantitative values of each index determined by the m *Monascus* strain (m = 10) and n evaluation indexes (n = 4). The original data were classified, as shown in Table 4.

#### 3.11.2. Standardization of Decision Matrix

The results are shown in Table 5.

#### 3.11.3. Entropy Measurement

P_ij_ reflects the relative performance of the evaluation object i under standard j, and the higher the P_ij_, the better the performance (Table 6). After the normalization of P_ij_, the information entropy and weight under each evaluation criterion can be calculated. Entropy *ej* measures the heterogeneity of performance scores on the criteria *j*. The higher the entropy, the greater the uncertainty. The weight *w*_*j*_ reflects the relative importance of the criteria *j* in the decision-making process, and the greater the weight, the greater the importance.

**Figure 19:**
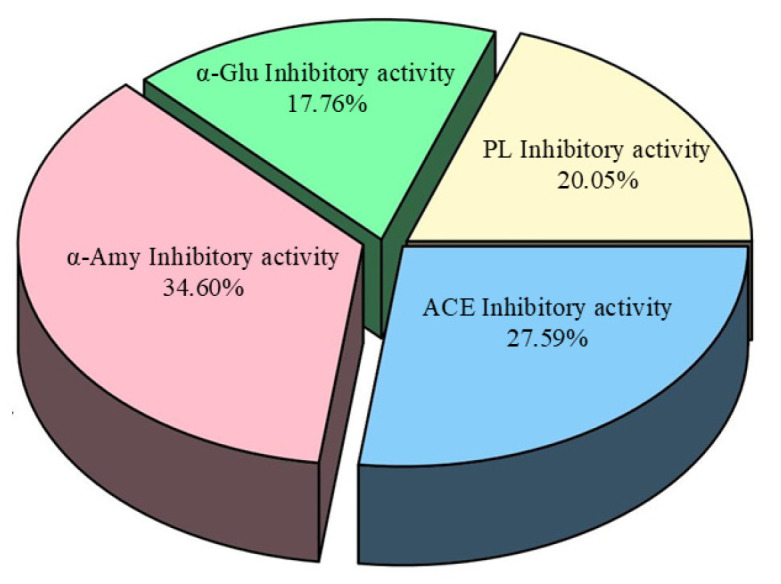
The weight of each evaluation criterion.

Entropy and entropy weights are shown in Table 7. The entropy weight of the four indicators is sequential as follows: α-Amy inhibitory activity (34.60%), angiotensin-converting enzyme inhibitory activity (27.59%), fatty enzyme inhibitory activity (20.05%), and α-glucosidase (17.76%). Among them, the entropy weight of α-Amy inhibition activity is the largest, and the comprehensive influence is the largest (Figure 19).

#### 3.11.4. Build a Weighted Standardized Decision Matrix

The decision matrix of the TOPSIS method is the same as in Table 4. SPSS Statistics 27 analysis software was used to analyze the data and calculate the weight. The results are shown in Table 7. The weighted standardized decision matrix is obtained by multiplying the weights of the standardized decision matrix and the evaluation indicators, and the results are shown in Table 8.

#### 3.11.5. Calculate Positive and Negative Ideal Solutions

Positive and negative ideal solutions provide an effective method to deal with multiple evaluation criteria when dealing with multi-criteria decision-making problems. Table 9 shows the positive and negative ideal solutions of each evaluation index. By comparing the distance between the evaluation standard value of each candidate *Monascus* strain and the two ideal solutions, different alternatives can be evaluated. The closer the actual value of the candidate strains is to the positive ideal solution, the more the candidate strains tend to perform optimally on multiple criteria. The farther away the value is from the negative ideal solution, the farther away it is from the worst performance of the alternative on multiple criteria.

#### 3.11.6. Calculation and Comprehensive Evaluation of Euclidean Proximity Degree

**Figure 20:**
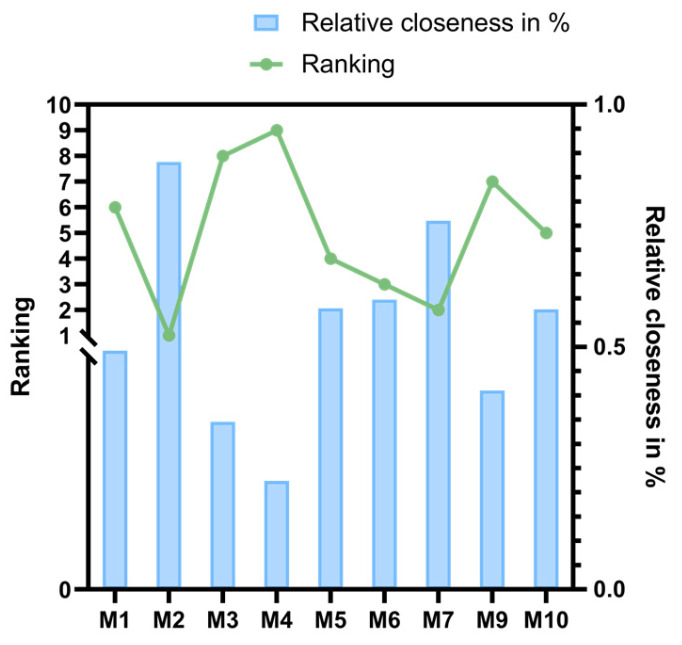
Comprehensive score and ranking of alternative *Monascus*.

In multi-attribute decision analysis, Euclidean approximation is the key index to measure the approximation between the solution and the ideal solution. In this study, the distances of each inhibition rate index of *Monascus* to the positive ideal solution and negative ideal solution were denoted as D+ and D−, respectively, and then the relative closeness was calculated. The closer the relative proximity was to one, the better the comprehensive evaluation of the *Monascus* strain, and the comprehensive ranking of the decrease in the high activity of each strain for the “three highs” was determined accordingly. Table 10 shows that the relative proximity degree of each *Monascus* strain ranged from 0.224 to 0.881, among which the relative proximity degree of the M2 strain was the highest, indicating that it is close to the best value (positive ideal solution) and far away from the worst value (negative ideal solution), with no obvious disadvantages. As shown in Figure 20, based on the comprehensive ranking, the M2 strain was identified as the strain with the largest decrease in high activity for the “three highs”.

### 3.12. The Hypotensive, Hypolipidemic, and Hypoglycemic Active Components of Monascue: MK and GABA

In this study, it was observed that the metabolites of *Monascus* demonstrated significant PL inhibitory activity, indicating the potential application of *Monascus* in lipid management. HPLC analysis confirmed the presence of MK in the metabolites of seven *Monascus* strains, a well-known hypolipidemic compound renowned for its efficacy in reducing LDL cholesterol, total cholesterol, and blood pressure in individuals with dyslipidemia [42]. The substantial quantity of MK found in the metabolites of the M2 *Monascus* strain is noteworthy, measuring at 18.078 mg/g and underscoring its promising role in lipid reduction strategies.

In this research endeavor, the hypoglycemic and hypotensive effects of *Monascus* metabolites were meticulously evaluated, uncovering substantial biological activity, particularly in terms of α-Glu inhibitory activity, α-Amy inhibitory activity, and ACE inhibitory activity. These outcomes hint at the promising potential of *Monascus* metabolites in mitigating blood glucose and blood pressure levels. Through meticulous HPLC analysis, the presence of GABA was ascertained in the metabolic byproducts of nine *Monascus* strains. GABA is recognized for its capacity to lower blood glucose and blood pressure via diverse mechanisms, including ACE activity inhibition, angiotensin production reduction, and neurotransmitter level modulation [43]. Notably, the content of GABA in the metabolites of the M2 strain was quantified at 5.137 mg/g.

In conclusion, the hypothesis posits that MK plays a pivotal role in the hypolipidemic activity attributed to *Monascus*, while GABA is suggested to function as the bioactive component responsible for lowering blood glucose levels and blood pressure.

## 4. Discussion

In this study, using *Monascus* products as research materials, microbiological isolation technology successfully yielded 14 representative strains. By meticulously observing the colony and microscopic morphology of these strains, a fundamental understanding of their species was established. Subsequently, the ITS sequences of these 14 isolate strains were sequenced and analyzed, leading to the identification of 10 as *Monascus* purpureus, designated as M1-M10. This procedure establishes a dependable foundation for species identification in future investigations.

Subsequently, in vitro enzyme inhibition assays were conducted to assess the inhibitory effects of the identified *Monascus* strains on PL, α-Glu, α-Amy, and ACE, aiming to investigate their potential biological activities in mitigating risk factors associated with the major cardiovascular diseases: hypertension, hyperglycemia, and hyperlipidemia. The findings indicated varying levels of inhibitory activity against PL, α-Glu, α-Amy, and ACE across all identified *Monascus* strains, suggesting their promising potential in the exploration and creation of products aimed at addressing hypotrichosis. Existing studies have shown that *Monascus* metabolites have been proven to have excellent effects in lowering blood lipids, blood sugar, and blood pressure [44,45]. In recent years, *Monascus* fermentation technology has made significant innovative progress in the field of fermented tea and fermented plant product food processing, resulting in the development of products such as *Monascus*-fermented soybeans (MFSs), *Monascus*-fermented Job’s tears (MFA), and *Monascus*-fermented ginseng. However, previous research has mainly focused on the evaluation of the “three highs” and lowering effects of fermented products, without a clear understanding of the precise source of these bioactivities. This study systematically evaluates the three bioactivities of lowering blood lipids, blood sugar, and blood pressure and provides *Monascus* strains with strong comprehensive lowering capabilities for the “three highs” and predictable optimal fermentation effects. This contributes to understanding the comprehensive role of *Monascus* strains in the prevention and treatment of cardiovascular diseases, diabetes, and other public health issues. Moreover, the multi-index evaluation system used in this study more comprehensively reflects the bioactivity of *Monascus* strains, avoiding the limitations of single indicators and enhancing the reliability and application value of the research results. It provides a more solid scientific basis for the development of functional foods and helps in the development of healthy foods that can simultaneously regulate blood lipids, blood sugar, and blood pressure.

Concurrently, to guarantee the safety of the chosen strains, we conducted CIT detection, targeting a potentially toxic substance that could be present in *Monascus* metabolites. Through HPLC analysis, we identified nine *Monascus* strains devoid of CIT, thereby excluding the M8 strain due to the presence of CIT, a critical measure in ensuring the food safety of the end product.

In this study, the EWM-TOPSIS combined with the comprehensive evaluation method was employed to allocate objective weights to each evaluation index, integrating multiple single indicators into a comprehensive evaluation value. This approach effectively mitigates the impact of subjective judgment on the outcomes and enhances the scientific and objective integrity of the evaluation system. The evaluation results using the EWM-TOPSIS method indicated that the M2 strain demonstrated the most substantial comprehensive inhibitory activity among all the tested strains. Its notable inhibitory effects on PL, α-Glu, α-Amy, and ACE activities warrant further consideration. The M2 strain exhibits outstanding activity, which may be attributed to its unique genetic background. Even within the same genus, genetic differences between different strains can lead to significant phenotypic variations, such as differences in metabolic pathways and enzyme activities. Additionally, the metabolic characteristics of the M2 strain may also be a key factor in its high activity. It is worth noting that cultural conditions have a significant impact on the metabolic activity of strains; we speculate that the cultural conditions in this experiment may be particularly conducive to the production of M2 strain metabolites. To gain a deeper understanding of the activity mechanism of the M2 strain, we aim to optimize the cultural conditions in subsequent studies to selectively enhance the corresponding activities of lowering blood sugar, blood lipids, and blood pressure and explore how these conditions affect its genetic and metabolic characteristics.

The M2 *Monascus* strain can be used as an additive in healthy foods for the development of functional foods that lower blood lipids, blood sugar, and blood pressure, such as health beverages and nutritional supplements, to help consumers regulate their blood lipids, blood sugar, and blood pressure. In terms of pharmaceutical applications, with its metabolites MK and GABA already being used as drug components, *Monascus*, as a food additive, not only enhances the functional aspects of foods in reducing the “three highs” but can also be used in the development of natural medicines or as adjuvant therapy in combination with conventional drugs to improve treatment efficacy and reduce side effects. In summary, the selected M2 *Monascus* strain with the activity of lowering the “three highs” has broad potential applications in functional foods and pharmaceuticals. It can not only provide consumers with more healthy options but also promote the development and innovation of the *Monascus*-fermented food industry.

In this study, the activity of the strain M5 was relatively low, which may have been influenced by culture environment factors such as temperature and pH value. The strain M5 might not adapt as well to the culture conditions used in this experiment as strain M2, thereby affecting its metabolic activity and overall viability. Furthermore, strain M5 may have differences in metabolic pathways compared to strain M2, leading to differences in the quantity or types of metabolites produced, which could directly impact the activity of the strain. At the same time, the genetic differences and variations in growth rate between strain M5 and strain M2 may also affect their activity.

The HPLC results revealed that M1, M2, M3, M6, M7, M8, M9, and other strains contained MK, while all *Monascus* strains except the M7 strains contained GABA. Variations were observed in the metabolite content and inhibitory activity among the M1–M10 *Monascus* strains. The primary objective of this study was to identify the *Monascus* strain with the most significant potential for reducing hyperlipidemia, hypertension, and hyperglycemia. Consequently, the M2 strain, which contained both MK and GABA, was chosen for its role in regulating blood lipid levels, blood pressure, and blood glucose. The active compounds MK and GABA discovered in this study are consistent with those reported in previous research [46], which confirms that these compounds are key factors in the inhibitory activity of the *Monascus* strain. The coexistence of MK and GABA in the M2 strain suggests its multifunctional efficacy in modulating these parameters through diverse mechanisms. This finding establishes a crucial scientific foundation for further exploration of these strains’ potential applications in developing multifunctional foods or pharmaceutical products targeting the management of hyperlipidemia, hypertension, and hyperglycemia.

The secondary metabolites of *Monascus*, including MK, *Monascus* red pigments, GABA, and others, have been proven to possess a variety of biological activities, such as lowering blood lipids and sugar, and anti-inflammatory, antioxidant, and antitumor effects. Evaluating the research on *Monascus* secondary metabolites can not only reveal their potential value in regulating physiological functions and preventing diseases but also provide a scientific basis for food safety and pharmaceutical development. The qualitative and quantitative analysis of *Monascus* secondary metabolites can establish product quality control standards to ensure product stability and consistency. At the same time, professional safety evaluations are crucial for ensuring the safety of *Monascus* secondary metabolites when used as food additives or pharmaceutical ingredients. Subsequent acute toxicity tests can be conducted to determine the toxic effects of *Monascus* secondary metabolites upon a single high-dose intake. Chronic toxicity studies of the long-term intake of *Monascus* secondary metabolites should also be carried out to assess their long-term effects on experimental animals, including carcinogenicity, teratogenicity, and reproductive toxicity. This includes preclinical safety assessments in animal models and, based on ensuring safety, human clinical trials to verify their safety and efficacy in the human body.

## 5. Conclusions

This study successfully screened a safe and efficient M2 *Monascus* strain that has significant effects on lowering blood lipids, blood sugar, and blood pressure. This discovery holds profound significance for the food sector. Firstly, by applying the M2 *Monascus* strain in food, it is possible to develop foods with specific health functions for lowering the “three highs” through the biosynthesis of important active substances such as Monacolin K and GABA. Secondly, the application of this strain can help reduce or replace the use of chemically synthesized drugs, lowering the risk of potential side effects and enhancing food safety. In addition, this discovery promotes innovation in the *Monascus*-fermented food industry, aiding in the functionalization and healthification of foods to address chronic diseases in humans. In summary, this research not only provides a scientific basis for the development of new functional foods but also contributes to the sustainable development of the food industry.

## 6. Patent

Patent Title: A Monascus Strain, Monascus Fermentation Products and Their Applications
Patent Application Number: 2025102051400
Patent Applicants: Southwest University, Chongqing Lisi Biotechnology Co., Ltd., Chongqing, China
Patent Inventors: Liu Chuling, Zhou Lang, Li Qiaoyu, Xu Li, Jia Yanan, Zhang Yuansong
Patent Application Status: Filed
Patent Application Date: 24 February 2025
Country of Patent: China

## Figures and Tables

**Table 1:** The strain was isolated from *Monascus* products.

Strain No.	*Monascus* Products	Provenance
M1	Hongqu glutinous rice wine grains (HGG)	Xiapu, Fujian Province, China
M2	HGG	Xuanwei, Yunnan Province, China
M3	HGG	Shouning, Fujian Province, China
M4	HGG	Minqing, Fujian Province, China
M5	HGG	Sanming, Fujian Province, China
M6	Red Kojic Rice	Gutian, Fujian Province, China
M7	Red Sufu	Fuan, Fujian Province, China
M8	HGG	Xingning, Guangdong Province, China
M9	HGG	Wenzhou, Zhejiang Province, China
M10	Red vinasse acid	Meizhou, Guangdong Province China
M11, M12, M13, M14	Outdoor natural substrate fermentation samples	Chongqing, China

**Table 2:** Colony morphology of isolated strains.

Strain No.	Upper	Reverse
M1	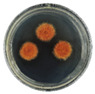	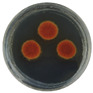
M2	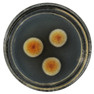	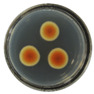
M3	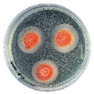	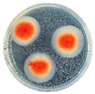
M4	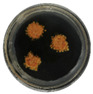	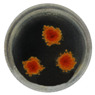
M5	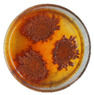	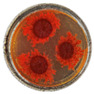
M6	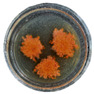	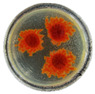
M7	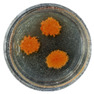	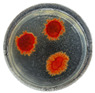
M8	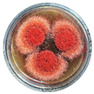	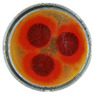
M9	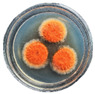	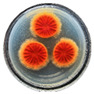
M10	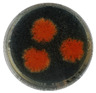	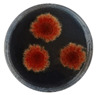
M11	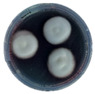	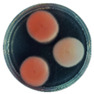
M12	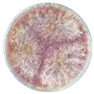	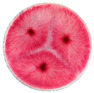
M13	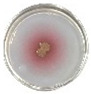	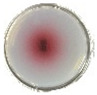
M14	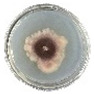	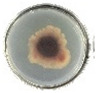

**Table 3:** Description of colony phenotypic characteristics of isolated strains.

Strain No.	Colony Morphology	Colony Color	Hyphal Morphology
M1, M4, M5, M6, M7, M10	Colony sprawl and margins are more regular, with no bulge.	Orange	Hyphae are short-flocculated and dense.
M2, M3	Colony with radioactive ditch and edge rules, no bulge.	Orange	The mycelium is short-flocculated, the center is dense, and the outer circle of mycelium is white.
M8, M9	Colonies are growing in sprawl, with more regular edges and no bulge.	Orange	Hyphae are short-flocculated and have more air-borne hyphae.
M11	Colonies grow in sprawl, with rounded edges and no bulge.	Pink	The mycelium is suede-like and dense.
M12	The colony is spreading and growing without bulge.	Pale purple-red	Hyphae are white or pale yellow, loose, spreading, and growing.
M13	Colony outward radiation, irregular edges, no bulge.	Dark purple	The mycelium is pale purple and is scarce and small.
M14	Irregular edge of the colony, slight uplift.	Dark red	The mycelium is dark orange or ink green and the mycelium is scarce.

**Table 4:** Original data of the detection results of MPLs with non-triple high activity.

No.	PL Inhibitory Activity (%)	α-Glu Inhibitory Activity (%)	α-Amy Inhibitory Activity (%)	ACE Inhibitory Activity (%)
M1	51.590	72.056	57.203	18.681
M2	81.903	96.938	72.215	60.440
M3	58.771	58.540	31.144	35.165
M4	39.256	65.103	33.153	0
M5	13.463	17.937	76.733	49.451
M6	54.604	61.147	53.601	60.440
M7	62.500	69.180	63.650	74.725
M9	57.806	67.383	47.774	17.582
M10	44.343	71.004	54.141	54.945

**Table 5:** Initializing the decision matrix.

No.	PL Inhibitory Activity	α-Glu Inhibitory Activity	α-Amy Inhibitory Activity	ACE Inhibitory Activity
M1	0.557186	0.685142	0.571707	0.250097
M2	1.000100	1.000100	0.900997	0.808932
M3	0.662111	0.514056	0.000100	0.470692
M4	0.376970	0.597130	0.044168	0.000100
M5	0.000100	0.000100	1.000100	0.661873
M6	0.601225	0.547055	0.492697	0.808932
M7	0.716596	0.648737	0.713123	1.000100
M9	0.648011	0.625991	0.364881	0.235389
M10	0.451298	0.671826	0.504542	0.735396

**Table 6:** Calculation results for indicator weight.

No.	PL Inhibitory Activity	α-Glu Inhibitory Activity	α-Amy Inhibitory Activity	ACE Inhibitory Activity
M1	0.111135	0.129513	0.124492	0.050306
M2	0.199478	0.189050	0.196197	0.162714
M3	0.132063	0.097172	0.000022	0.094678
M4	0.075190	0.112876	0.009618	0.000020
M5	0.000020	0.000019	0.217777	0.133133
M6	0.119919	0.103410	0.107287	0.162714
M7	0.142931	0.122631	0.155286	0.201166
M9	0.129251	0.118332	0.079455	0.047348
M10	0.090015	0.126996	0.109867	0.147922

**Table 7:** Reduce the weight coefficient of the three high indicators.

Criterion	Entropy	Coefficient of Variation	Weight
PL inhibitory activity	0.929108	0.070892	0.200463
α-Glu inhibitory activity	0.937191	0.062809	0.177606
α-Amy inhibitory activity	0.877626	0.122374	0.346041
ACE inhibitory activity	0.902434	0.097566	0.275890

**Table 8:** Weighted normalization matrix.

No.	PL Inhibitory Activity	α-Glu Inhibitory Activity	α-Amy Inhibitory Activity	ACE Inhibitory ACTIVITY
M1	0.111695	0.121685	0.197834	0.068999
M2	0.200483	0.177623	0.311782	0.223176
M3	0.132729	0.091299	0.000035	0.129859
M4	0.075569	0.106054	0.015284	0.000028
M5	0.000020	0.000018	0.346076	0.182604
M6	0.120524	0.097160	0.170493	0.223176
M7	0.143651	0.115219	0.246770	0.275918
M9	0.129902	0.111180	0.126264	0.064942
M10	0.090469	0.119320	0.174592	0.202888

**Table 9:** Ideal positive and negative measures.

	**PL Inhibitory Activity**	**A-Glu Inhibitory Activity**	**α-Amy Inhibitory Activity**	**ACE Inhibitory Activity**
Positive Ideal Solution	0.200483	0.177623	0.346076	0.275918
Negative Ideal Solution	0.000020	0.000018	0.000035	0.000028

**Table 10:** Index positive and negative ideal solution calculation results.

No.	D+	D−	Score
M1	0.275324	0.266751	0.492093
M2	0.062910	0.467666	0.881430
M3	0.391305	0.206882	0.345848
M4	0.454163	0.131087	0.223984
M5	0.283614	0.391253	0.579748
M6	0.215589	0.320639	0.597952
M7	0.130330	0.413394	0.760302
M9	0.319726	0.222203	0.410022
M10	0.224148	0.306654	0.577718

## Data Availability

The original contributions presented in the study are included in the article, further inquiries can be directed to the corresponding author.
